# Detection of *Alcanivorax* spp., *Cycloclasticus* spp., and *Methanomicrobiales* in water column and sediment samples in the Gulf of Mexico by qPCR

**DOI:** 10.1007/s11356-019-06551-7

**Published:** 2019-11-03

**Authors:** Edna L. Hernández-López, Jahaziel Gasperin, Johanna Bernáldez-Sarabia, Alexei F. Licea-Navarro, Abraham Guerrero, Marcial Leonardo Lizárraga-Partida

**Affiliations:** Centro de Investigación Científica y de Educación Superior de Ensenada, Carretera Ensenada-Tijuana 3918, zona Playitas, 22860 Ensenada, Baja California México

**Keywords:** Petroleum biodegradation, Southern Gulf of Mexico, *Alcanivorax*, *Cycloclasticus*, *Methanomicrobiales*, Hydrocarbonoclastic bacteria, Oil-degrading bacteria, Deep sea

## Abstract

Water column and sediment samples were collected in the southern Gulf of Mexico (GoMex) during 3 oceanographic cruises: XIXIMI-04 (September 2015), XIXIMI-05 (June 2016), and XIXIMI-06 (August 2017). DNA that was extracted from the samples was analyzed by qPCR to detect and quantify bacterial groups that have been reported to metabolize alkanes (*Alcanivorax*) and aromatic hydrocarbons (*Cycloclasticus*) and are involved in methane production (*Methanomicrobiales*)*.* The results were then analyzed with regard to the water masses that are currently detected in the GoMex. Generally, we observed a decrease in the proportion of *Alcanivorax* and a rise in those of *Cycloclasticus* and *Methanomicrobiales* in samples from the surface to deep waters and in sediment samples. Scatterplots of the results showed that the relative abundance of the 3 groups was higher primarily from the surface to 1000 m, but the levels of *Cycloclasticus* and *Methanomicrobiales* were high in certain water samples below 1000 m and in sediments. In conclusion, oil-degrading bacteria are distributed widely from the surface to deep waters and sediments throughout the southern GoMex, representing a potential inoculum of bacteria for various hydrocarbon fractions that are ready for proliferation and degradation in the event of an oil spill from the seafloor or along the water column.

**Electronic supplementary material:**

The online version of this article (10.1007/s11356-019-06551-7) contains supplementary material, which is available to authorized users.

## Introduction

The Gulf of Mexico (GoMex), a semi-enclosed basin, is an important area for fisheries, tourism, and the oil industry in the USA and Mexico. According to the US Energy Information Administration (2018), the mean oil production from 2010 to 2018 in the US area of the northern GoMex was estimated to be 1.6 million barrels per day (1 barrel of oil = 159 L).

The water masses in the GoMex from the Atlantic Ocean and Caribbean Sea have been characterized with regard to salinity, temperature, and dissolved oxygen, in the Yucatan channel and inside of the GoMex (Rivas et al. 2005). From the surface to a depth of approximately 1000 m, the water masses have higher phytoplankton productivity (normally corresponding to a fluorescence maximum < 100 m), followed by 18 °C Sargasso Sea water (200–400 m), low-oxygen water mass (400–600 m) that is characteristic of Tropical Atlantic Central Water (TACW), and Antarctic Intermediate Water (AAIW; 600–900 m). Below 1000 m, the GoMex is filled with North Atlantic Deep Water (NADW), characterized by salinity maximum (35.0 psu), low temperature (4 °C), and high (> 5 ml/L) dissolved oxygen concentration (Rivas et al. 2005). River systems such as the Mississippi-Atchafalaya in the north and the Coatzacoalcos, Papaloapan, and Grijalva-Usumacinta in the south significantly influence the ecology of the GoMex (Lizárraga-Partida 1996).

However, the principal hallmark of the Gulf is the presence of oil and natural gas (primarily methane) seeps, in the deep and coastal areas of Mexico and the USA, from 30 to 3000 m. According to Joye et al. (2016), at least 22,000 natural seeps have been identified in the entire Gulf, approximately 1000 of which are experiencing intense activity and are persistent. Estimates of natural seepage rates for oil from GoMex natural seeps vary between 1500 and 3800 barrels per day (Joye et al. 2016), representing an important supply of organic matter that can be used as a carbon source by microorganisms, especially bacteria. Consequently, the entire ecosystem (water column and sediments) represents a food web that is highly influenced by these oil and gas seeps, containing bacteria that are directed toward hydrocarbon degradation.

These seeps have been a valuable indication of oil reservoirs, and since the drilling of the first oil well on the coast of Louisiana in 1937 (Pratt et al. 1997), the GoMex has become one of the most promising regions for the oil industry. Yet, the discovery of new reservoirs has increased the risk of accidental oil spills. The massive oil spills of the Ixtoc I blowout (3.4 million barrels) in the southern GoMex in 1979 (Soto et al. 2014) and the Deepwater Horizon accident (DWH)—in which 5 million oil barrels and 250,000 metric tons of gas, primarily methane, were released into the northern GoMex in 2010 (Joye 2015)—have demonstrated the difficulty in evaluating large-scale environmental disturbances and assessing the return to normal ecological conditions, without previous baseline studies.

Studies that were conducted 1 year after the DWH spill reported that the hydrocarbon concentrations in the water column remained low (Yergeau et al. 2015). DWH studies also detected an unrecognized diversity of closely related taxa that are affiliated with *Cycloclasticus*, *Colwellia*, and *Oceanospirillaceae*, which are usually not observed in natural hydrocarbon seeps (Kleindienst et al. 2016). In beach sands that were impacted by the DWH accident, several components such as *n*-alkanes were reported; based on this, it was established that the gammaproteobacteria *Alcanivorax* spp. could be used as a microbial indicator of the early stages of oil hydrocarbon degradation (Kostka et al. 2011).

Oil-degrading bacteria (ODB) that use saturated hydrocarbons as their carbon source, such as *Alcanivorax* spp., *Marinobacter* spp., and *Oleiphilus* spp., and those that metabolize PAHs, such as *Cycloclasticus* spp. and *Neptumonas* spp. (Head et al. 2006), are normally detected in marine habitats. Smith et al. (2013) reported the presence of ODB that harbor the alkane hydroxylase gene (*Alcanivorax-Marinobacter*) in the waters of the northern Gulf of Mexico, immediately prior to the DWH accident. Once an accidental oil spill occurs in marine systems, ODB flourish and become dominant members of the microbial community (Teramoto et al. 2009; Vila et al. 2010). According to Hazen et al. (2010), cold-adapted marine microorganisms respond rapidly to hydrocarbon exposure during the DWH accident, within hours to days.

There are significant amounts of the γ-proteobacteria *Alcanivorax* spp. and *Cycloclasticus* spp. in the marine environment. *Alcanivorax* spp. are considered an obligate hydrocarbon degrader, primarily aliphatic hydrocarbons (Joye et al. 2014). Since the isolation of the first member of the genus *Alcanivorax* (*A. borkumensis*), more than 250 bacteria that are associated with *Alcanivorax* have been isolated or detected, based on 16S rRNA gene sequences, in various marine habitats (Head et al. 2006). Members of the genus *Cycloclasticus*, such as *C. pugetii*, were first isolated in Puget Sound sediments (Dyksterhouse et al. 1995); since then, Maruyama et al. (2003) have shown that along the oil-polluted Mikuni coast, ODB that are phylogenetically related to the species *C. pugetii* are the predominant heavy-oil decomposers. Further, bacteria that are similar to *C. pugetii* have been isolated in samples from the GoMex (Geiselbrecht et al. 1998).

The order *Methanomicrobiales* includes genera that are involved in the production of methane under anaerobic conditions from various carbon sources, such as aliphatic hydrocarbons (Zhou et al. 2012). Methane from natural seeps and unknown sources has been detected from deep to surface waters in the GoMex, influencing the microbial community structure (Rakowski et al. 2015). Although no studies have described the distribution of *Methanomicrobiales* in the GoMex, perhaps due to the high dissolved oxygen concentration along the water column, the microbial production of methane, usually reported as an anaerobic process, can occur in well-oxygenated water column samples from a freshwater lake (Grossart et al. 2011).

Current and future oil activities in the Mexican waters of the GoMex will increase the risk of an accident, necessitating baseline ecological studies, including an assessment of the distribution of hydrocarbon-related **γ**-proteobacteria genera, such as *Alcanivorax* and *Cycloclasticus*, and the *Archaea* order *Methanomicrobiales.* In this study, we generated baseline data on these groups by qPCR analysis of 30 sediment samples and 1081 water column samples that were collected during 3 oceanographic cruises in the southern GoMex.

## Materials and methods

### Sample collection

**Fig. 1:**
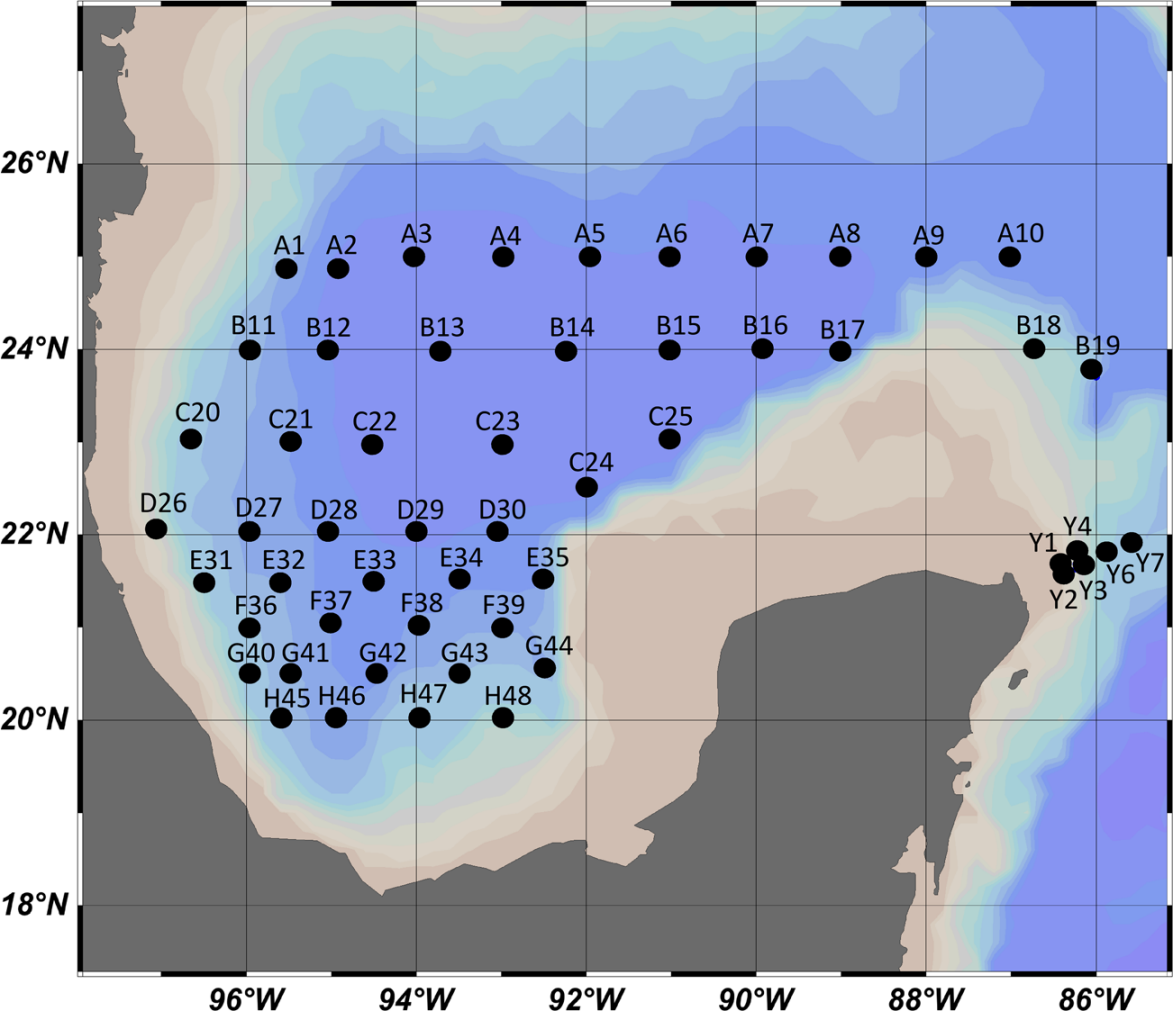
Sampling locations in the GoMex for the three oceanographic cruises. Some locations were sampled for water and/or sediment in all cruises (Table S1). Continental platform is indicated in brown

Samples were collected during 3 oceanographic cruises (Fig. 1), onboard the research vessel Justo Sierra (Universidad Nacional Autónoma de México, UNAM): XIXIMI-04 (XIX-04) in September 2015, during the rainy season; XIXIMI-05 (XIX-05) in June 2016, during the end of the dry season; and XIXIMI-06 (XIX-06) in August 2017, also during the rainy season. The first XIXIMI line of sampling locations was located at 25° N, and the nearest XIXIMI sampling location to the oil spill areas in the USA was located at 25° N and 88° W, approximately 350 km south (Fig. 1) of the DWH platform and the Taylor Energy Co. platform.

A total of 1081 water samples were collected at 54 sampling locations (Fig. 1) using 12 × 20 L Niskin bottles in an oceanographic rosette system that could be closed remotely at selected depths of the water column by real-time monitoring using a Seabird^TM^ CTD, with probes for conductivity, depth, temperature, fluorescence, and dissolved oxygen. The selected depths were related to the water masses that have been reported for the GoMex (Rivas et al. 2005): the fluorescence maximum (50–100 m), 250 m, 300 m, the oxygen minimum, (± 400 m: TACW), 600 m, 800 m, 1000 m (AAIW), 1200 m, 2000 m, 2500 m (NADW), and the bottom (10 m above the sea floor). Two liters at each depth was passed through a 0.22-μm Sterivex™ filter (EMD Millipore, Burlington, MA, USA) immediately after sample collection. A total of 30 sediment samples were collected at selected points on all cruises (Table S1) using a Reineck box corer. A subsample from the surface layer (~ 10 cm depth) was taken using 10-mL sterile syringes; stored at 4 °C; transported at 4 °C to CICESE in Ensenada B.C., Mexico; and stored at − 20 °C until DNA extraction.

### DNA extraction

DNA was extracted using the Wizard Genomics DNA™ purification kit (Promega Corportion, Madison, WI, USA) as per the instructions for Gram-negative bacteria, with the following modification: nuclei lysis solution was added directly to the Sterivex™ filter, capped with handmade plugs, and placed in a hybridization oven for incubation with smooth and steady rotation (80 °C, 5 min). The solution was collected in a 1.5-mL Eppendorf tube, and the proteins and DNA were precipitated per the manufacturer’s instructions. DNA was suspended in 50-μL nuclease-free water. The PowerMax™ Soil (MoBio, Carlsband, CA, USA) DNA isolation kit was used for sediment samples. The quality and concentration of the extracted DNA were measured on a NanoDrop Li™ (Thermo Fisher Scientific Inc., Waltham, MA, USA). DNA samples were stored at − 80 °C until analysis.

### Primer selection

Primers were designed to amplify a fragment of the 16S-SSU-rDNA gene in the hydrocarbon-degrading prokaryotes *Alcanivorax*, *Cycloclasticus*, and *Methanomicrobiales*. The primer sequences are listed in Table 1.

**Table 1:** Primer pairs for ODB in the real-time quantitative PCR (qPCR) assays

Target (16S SSU rDNA)	Name	Primer sequence 5′-3′	Size (bp)	Reference
*Alcanivorax*	Alcvx-464F	GAGTACTTGACGTTACCTACAG	211	Kostka et al. 2011
	Alcvx-675R	ACCGGAAATTCCACCTC		
*Cycloclasticus*	Cyc-467f	AACCTTAGGCCCTGACGT	128	Gutierrez et al. 2015
	Cyc-577r	TGTTTAACCGCCTACGCG		
*Methanomicrobiales*	MMB749F	TYCGACAGTGAGGRACGAAAGCTG	84	Yu et al. 2005
	MMB832R	CACCTAACGCRCATHGTTTAC		

For *Alcanivorax*, the Alcvx-464F/Alcvx-675R primer pair (Kostka et al. 2011) was used, which amplified a 211-bp product with a dissociation temperature (Tm) of 79.7 °C ± 0.6 °C. For *Cycloclasticus*, the Cyc-467F/Cyc-577r pair (Gutierrez et al. 2015) amplified a 128-bp product with a Tm of 83.9 °C ± 0.4 °C. An 84-bp product with a Tm of 74.9 °C ± 0.4 °C was obtained for *Methanomicrobiales* with MMB749F/MMB832R primer pair (Yu et al. 2005). The primers in this study were evaluated by gel electrophoresis (data not shown) and qPCR using a standard DNA template.

### Construction standard curve

Standard plasmids were constructed with the product of each pair of primers, amplified by PCR from a mixture of genomic DNA from a previous oceanographic cruise. Products were cloned using the TOPO-TA™ cloning kit (Invitrogen™, Carlsbad, CA, USA), and the resulting plasmids were purified by low-scale alkaline lysis and sent to SeqXcel Inc. (San Diego, CA, USA) for sequencing with M13F/R primers.

The optimal alignment temperature for each pair of primers was determined. The PCR products, run through a temperature gradient (45–60 °C Δ 3 °C), were analyzed on a 2% agarose gel. The temperature that allowed the most amplification of a specific product was selected for the subsequent assays. The PCR reaction (25 μL) contained 1 U of GoTaq™ DNA polymerase (Promega Corportion, Madison, WI, USA), 1× GoTaq™ Buffer, 1.5 mM MgCl_2_, 0.2 mM dNTPs, 500 nM of each primer, and 1 × 10^6^ copies of template (specific standard plasmid). An 8-point standard curve (2.40 × 10^7^ – 3.07 × 10^2^ copies) was generated for each product from the plasmids, for which the product was amplified with M13F/R primers, purified using the Purelink™ PCR purification kit (Invitrogen™, Carlsbad, CA, USA), quantified by spectrophotometry at 260 nm, and serially diluted (1:5) with nuclease-free water. Each point was run in triplicate by qPCR, as described below.

Standard curves were constructed for the 3 genes, corresponding to each taxon. For *Alcanivorax* (*y* = − 3.53*x* + 38.39, *R*^2^ = 0.99, E = 91.87%), *Cycloclasticus* (*y* = − 3.35*x* + 35.70, *R*^2^ = 0.99, E = 98.45%), and *Methanomicrobiales* (*y* = − 3.52 + 36.79, *R*^2^ = 0.99, E = 92.32%) the standard curves were linear over 8 orders of magnitude, ranging from 10^2^ to 10^8^ gene copies per μL.

### Quantification by qPCR

The 10-μL reaction comprised 5-μL SYBR Green master mix™ (Applied Biosystems, Foster City, CA, USA), 2.5-μL nuclease-free water, 0.25 μL of each primer (10 nM), and 2 μL of extracted DNA. The program was as follows: an initial cycle at 95 °C for 5 min; 40 cycles of 95 °C for 30 s, optimal annealing temperature for 30 s, and extension at 72 °C for 30 s; and dissociation from 60 to 95 °C in increments of 1% (~ 0.4 °C). The assays were run on a 7500 Real-Time PCR system™ (Applied Biosystems, Foster City, CA, USA).

The number of copies/L or copies/kg, for water or sediment, respectively, was determined based on the obtained standard curves*.* All depths were normalized to their highest value in each sampling location. The normalization was performed by dividing the value of the number of copies at each depth by the highest value at their respective sampling location, obtaining a relative abundance of 0 to 1.

The qPCR-normalized values for the 3 groups of ODB were plotted as relative abundance using Ocean Data View (Schlitzer 2017), according to the results for the water masses and sediment samples from the XIX-04, XIX-05, and XIX-06 cruises.

## Results

**Fig. 2:**
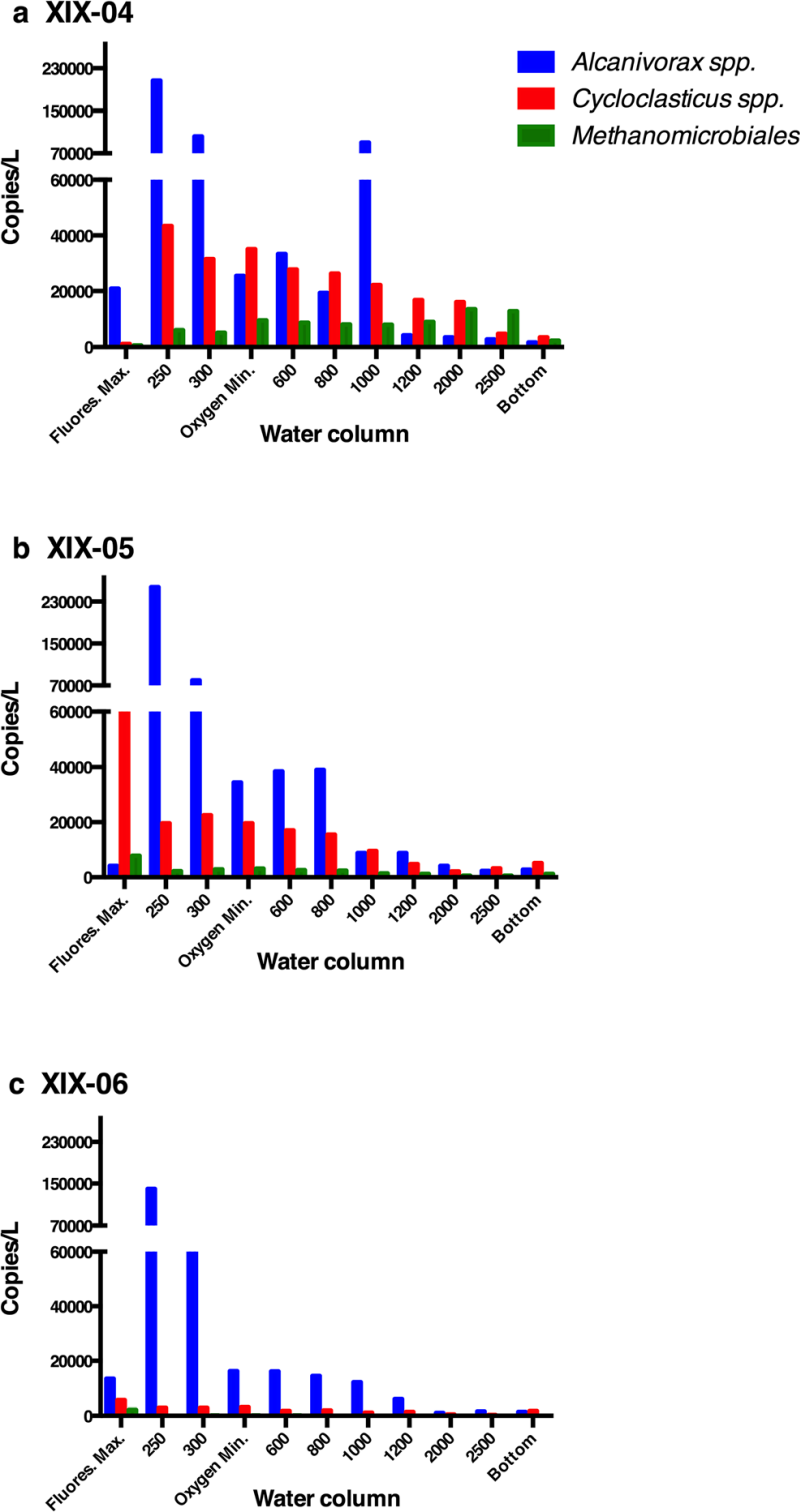
Mean concentration of number of copies /L at each depth for *Alcanivorax*, *Cycloclasticus*, and *Methanomicrobiales.***a** Cruise XIX-04. **b** Cruise XIX-05. **c** Cruise XIX-06

In the qPCR analysis, 1081 water samples from the southern GoMex were measured by the number of copies/L (Fig. 2a–c). On all cruises, the water samples had concentrations that ranged from 10^1^ to 10^5^ for *Alcanivorax*, 10^1^ to 10^4^ for *Cycloclasticus*, and undetectable to 10^4^ for *Methanomicrobiales.* On all cruises, *Alcanivorax* spp. were the predominant group from 250 to 1000 m. The samples at 250 m had a maximum concentration of 10^5^ copies per liter for *Alcanivorax*. *Cycloclasticus* and *Methanomicrobiales* levels were lower in water samples from the XIX-06 cruise. In sediment samples, the concentration of *Alcanivorax* ranged from undetectable to 10^6^ copies per kg, compared with undetectable to 10^7^ for *Cycloclasticus* and *Methanomicrobiales*. Based on the ODV maps, the distribution of the relative abundance of the 3 groups differed according to the depth of sampling and in sediment.

**Fig. 3:**
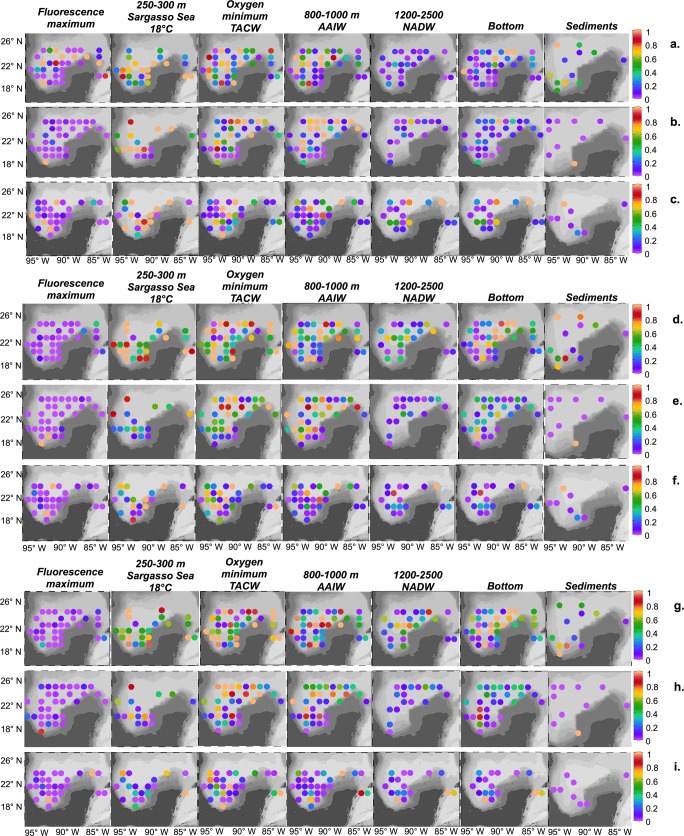
Spatial distribution of oil-degrading bacteria. Relative abundance in the water column and sediments for **a***Alcanivorax* spp. XIX-04, **b***Alcanivorax* spp. XIX-05, **c***Alcanivorax* spp. XIX-06, **d***Cycloclasticus* spp. XIX-04, **e***Cycloclasticus* spp. XIX-05, **f***Cycloclasticus* spp. XIX-06, **g***Methanomicrobiales* spp. XIX-04, **h***Methanomicrobiales* spp. XIX-05, and **i***Methanomicrobiales* spp. XIX-06

Figure 3a–i shows the ODV representation of the relative abundance, from 0 to 1, for the 3 groups of ODB at selected water depths and sediments. We selected ODB that were representative degraders of aliphatic hydrocarbons (*Alcanivorx* spp.) and aromatic hydrocarbons (*Cycloclasticus* spp.) and a group of bacteria that were related to methane metabolism (order *Methanomicrobiales*). Each point represents a sampling location, and the scale of the colors corresponds to the relative abundance of each group of ODB in the water mass and sediment samples.

**Fig. 4:**
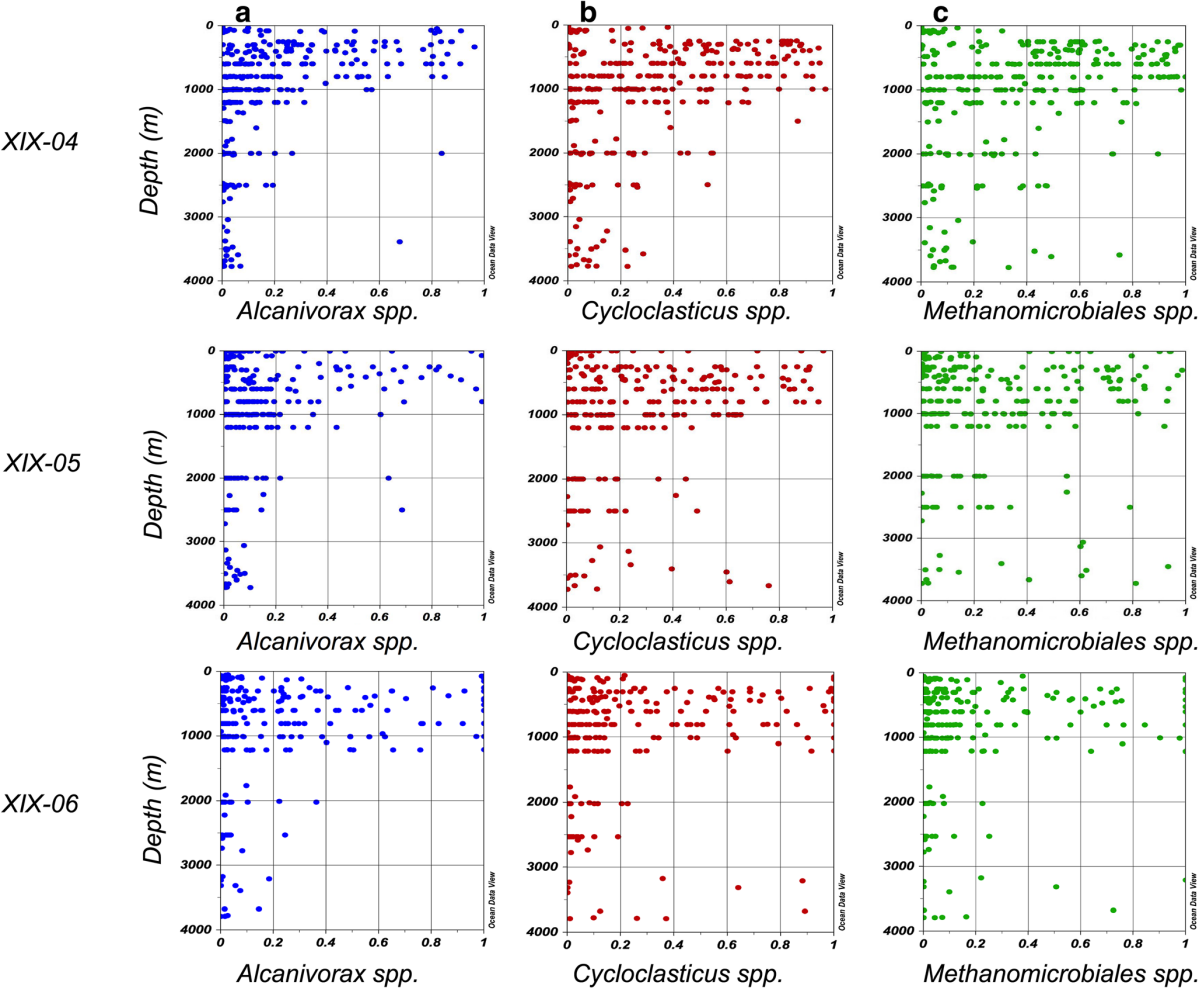
Scatterplot of oil-degrading bacteria. Relative abundance in the water column for XIX-04, XIX-05, and XIX-06. **a***Alcanivorax* spp. **b***Cycloclasticus* spp. **c***Methanomicrobiales* spp.

The relative abundance of ODB groups was distributed widely along the water column but at low numbers; nevertheless, ODB were present throughout the water column and sediments on all cruises. ODB were more abundant from 250 to 1000 m on all cruises (Figs. 3 and 4), notably in the water mass samples of 18 °C Sargasso Sea water (250–300 m) and TACW (400–600 m) and at 800–1000 m, corresponding to AAIW. The near-bottom water samples yielded more locations with a higher relative abundance than NADW (1200 to 2500 m).

By sampling depth, for all cruises, *Alcanivorax* increased from 250 to 1000 m, declined from 1200 to 2500 m, and rose at certain locations in near-bottom waters. Several locations had a higher relative abundance in XIX-04 versus XIX-5 and XIX-06. The genus *Cycloclasticus* had a similar distribution as *Alcanivorax* but with greater abundance at 1200–2500 m and in the bottom samples. Samples with high relative abundance were more frequent in XIX-04 compared with those in XIX-05 and XIX-06. The order *Methanomicrobiales* had a similar distribution as *Cycloclasticus*, with an increase at 1200–2500 m and in the bottom samples, and higher relative abundance in XIX-04 versus XIX-05 and XIX-06. Based on the geographic distribution of the ODB groups in the water samples, most of the high-abundance points were observed north of 22 °N.

For sediments, the relative abundance of each group of ODB was greater in samples from XIX-04 (Fig. 3a, d, g) with respect to XIX-05 and XIX-06. The sampling station in the seep area of Campeche Canyon (92° W/20° N), which was sampled on all cruises, had greater abundance of the 3 groups of ODB only in XIX-05.

The distribution of relative abundance of each group of ODB in the water mass samples was also analyzed by scatterplot using the ODV program, in which the results from all stations and depths were plotted simultaneously, ranging from 0 to 1 (Fig. 4). From the fluorescent maximum to 1000 m, most values were between 0 and 0.4, but many locations registered values of between 0.4 and 1. In water mass samples deeper than 1000 m (NADW), most values for *Alcanivorax* were between 0 and 0.2, compared with 0.2 to 0.8 for *Cycloclasticus* and *Methanomicrobiales*, showing that the latter groups predominate in this water mass, especially for samples at 10 m above the bottom, at depths of approximately 3000 m.

**Fig. 5:**
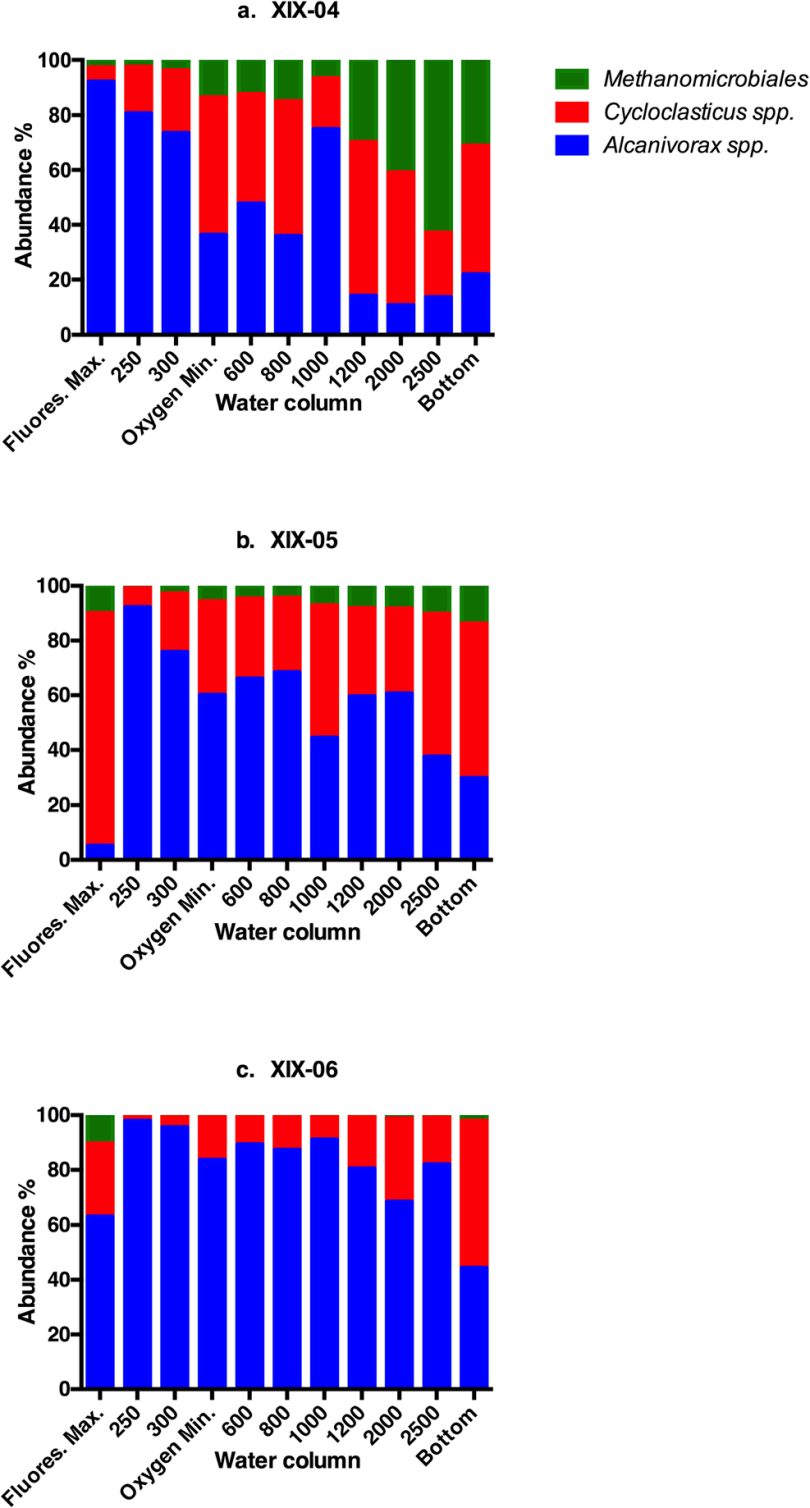
Percentage of the three groups of ODB at select water depths for XIX-04 (**a**), XIX-05 (**b**)**,** and XIX-06 (**c**)

Relative abundance, expressed as percentage, is plotted in Fig. 5a–c. *Alcanivorax* spp. predominated at surface levels, and *Cycloclasticus* spp. increased toward the deepest levels. These results suggest that *Alcanivorax* and *Cycloclasticus* abound at various levels of the water column compared with *Methanomicrobiales*. These trends were similar for all cruises. However, the percentages for *Methanomicrobiales* decreased considerably in XIX-06.

## Discussion

The ubiquity of ODB, represented in this study by the γ-proteobacteria *Alcanivorax* spp. and *Cycloclasticus* spp. and the *Archaea* order *Methanomicrobiales*, indicates that water mass and sediment from the southern GoMex harbor a community of bacteria that can act as an inoculum to respond rapidly to an oil input. Our results show that the baseline concentration of these ODB ranges from undetectable to 10^6^ copies per liter in the water column and undetectable to 10^7^ copies per kg for sediment. The concentration of ODB in the water samples was low compared with total direct cell counts of approximately 10^4^ cells per mL, as reported by Hazen et al. (2010) for the northern GoMex, in water samples from 600 to 1400 m.

During the Deepwater Horizon (DWH) blowout, Hazen et al. (2010) recorded an increase in γ-proteobacteria and hydrocarbon biodegradation rates at 5 °C, faster than expected, at the hydrocarbon plume between 1099 and 1219 m. Genomic studies performed by this group have indicated that plume samples have greater cell densities but lower diversity, predominated by γ-proteobacteria of the order *Oceanospirillales*. Camilli et al. (2010) reported higher methane and aromatic hydrocarbon concentration at depths at which the oil plume resided; thus, these hydrocarbons were the main available carbon source for the γ-proteobacteria that were detected.

By 16S rRNA and pyrosequencing analysis, Yang et al. (2016) monitored the succession of microbial communities before, during, and after the DWH blowout. They found that the pre-spill baseline community composition comprised primarily the γ-proteobacteria families *Oceanospirillales* and *Alteromonadales*. Samples in the oil plume during the spill were predominated by the so-called DWH *Oceanospirillales* cluster, which includes the genera *Cycloclasticus* and *Colwellia*. These genera were also detected 5 days after the spill and became dominant in the oil plume 2 weeks later (Redmond and Valentine 2012). Post-spill water column samples that were collected after 4 months from where the oil plume was located contained detectable but low levels of DWH *Oceanospirillales*. However, alkane degraders, such as *Alcanivorax*, remained detectable at a rate of < 1%, and PAH degraders, such as *Cycloclasticus*, were identified at rates of 5%, based on pyrosequencing reads in the water column samples, potentially representing the baseline ODB of the area (Yang et al. 2016).

The greater relative abundance of the ODB from 250 to 1000 m might be related to marine snow, which in GoMex could become marine oil snow, an event that occurred during the DWH oil spill. Marine snow aggregates (> 0.5 mm) that resulted from zooplankton activity in surface waters and sinking to deep waters have been considered to be a hot spot for microbial communities and an important event for oil sedimentation in the GoMex (Joye et al. 2016). From the surface water to deep water, the density increased constantly in the GoMex (Ochoa, CICESE personal communication), a variable that could slow the sedimentation of seep oil snow aggregates at greater depths—a hypothesis that could explain the peak relative abundance of the ODB, as detected by qPCR, from 250 to 1000 m on all XIXIMI cruises.

The distribution of ODB groups could also be attributed to the concentration of aliphatic or aromatic hydrocarbons in the water column. *Alcanivorax* spp., the levels of which were higher from 250 to 1000 m, might have been associated with alkane concentration, which migrates to surface waters. Camilli et al. (2012) reported that C 2+, C 3+, and C 4+ aliphatic hydrocarbons peaked in concentration at surface waters during the DWH spill. Aromatic hydrocarbons that are oxidized by *Cycloclasticus* tend to be more abundant in deep waters, as reported during the DWH blowout (Camilli et al. 2012); this cold-tolerant genus also has the advantage of being able to grow at temperatures between 4 and 20 °C (Coulon et al. 2007). These characteristics could explain the tendency of *Cycloclasticus* to predominate at depths of greater than 1000 m in the GoMex.

It is difficult to explain the presence of *Methanomicrobiales* in the water column in the GoMex samples, because these bacteria require anaerobic conditions to metabolize organic compounds to methane. Rakowski et al. (2015) reported that the methane in the aphotic zone of the GoMex originated from methane seeps but that the source of supersaturated concentrations of methane, relative to the atmosphere, in the photic zone (0–200 m), primarily in the deep-chlorophyll maximum (± 83 m), is unknown. Grossart et al. (2011) indicated that despite the accepted paradigm that methanogenesis takes place in anoxic environments, “methane production could and did occur” in well-oxygenated water masses. This phenomenon is important for the GoMex, where the oxygen concentration is between 2.5 and 5 ml/L (Rivas et al. 2005), with higher values toward deep waters, where the abundance of *Methanomicrobiales* is greater.

## Conclusion

The presence of ODB throughout the water column in the southern GoMex, from near-surface to near-seafloor waters, including sediments, indicates that this area could react quickly to an accidental oil spill by increasing the concentration of ODB in the plume of spilled oil, as observed during the 2010 New Horizon blowout (Hazen et al. 2010). Therefore, the existing data suggest that the GoMex harbors ODB that could respond to a massive oil input and that low temperatures (4 °C) or oxygen concentrations in deep waters 1000 m) are not limiting factors. Nevertheless, nutrient concentrations and the use of chemical oil dispersants remain to be evaluated in a massive oil spill.

## Electronic supplementary material

ESM 1 (XLSX 48 kb)
